# Assessing the knowledge of expectant mothers on mother–to-child transmission of viral hepatitis B in Upper West region of Ghana

**DOI:** 10.1186/s12879-017-2490-x

**Published:** 2017-06-12

**Authors:** Frederick Dun-Dery, Martin Nyaaba Adokiya, Williams Walana, Ernestina Yirkyio, Juventus B. Ziem

**Affiliations:** 1 Department of Community Health, School of Allied Health Sciences, University for Development Studies (UDS), Tamale, Ghana; 2 Department Clinical Microbiology, School of Medicine and Health Sciences, UDS, Tamale, Ghana; 3 Clinical Laboratory Department, Tamale Teaching Hospital, Tamale, Ghana

**Keywords:** Knowledge, Hepatitis B, Mother-to-child transmission, Expectant mothers, Ghana

## Abstract

**Background:**

Viral Hepatitis B is of a major public health concern globally, especially in developing countries. Expectant mothers’ knowledge of Mother-To-Child Transmission (MTCT) of the disease is significant in preventing the spread from an infected mother to her child. This study sought to assess the expectant mothers’ knowledge of Mother-To-Child Transmission of viral hepatitis B in the Wa Municipality and Lawra District of Upper West Region, Ghana.

**Methods:**

A descriptive cross-sectional study with a multi-stage sampling technique was employed to select a total of 450 study respondents (expectant mothers), and a semi-structured questionnaire was used for the data collection. Respondents were interviewed using face-to-face interview technique.

**Results:**

Majority (54.0%) of the respondents were aged between 25 and 35 years and the results were similar in both districts. Overall, 62.4% (281/450) of the respondents had at least Junior High level education, and 76.2% (343/450) were multigravida. Educational levels among respondents in the two areas were above 50.0% and considered relatively high. Respondents’ general knowledge of hepatitis B infection and disease was 46.0% (208/450). However, there was a slight difference between the two districts (40.1% in Lawra District and 51.6% in Wa Municipality). The overall knowledge level on MTCT of viral hepatitis B among the respondents was 34.7% (156/450): the Wa Municipality recorded higher knowledge (43.3%) compared to 24.8% in Lawra District.

**Conclusion:**

The knowledge level of the expectant mothers on MTCT of viral hepatitis B is relatively low in Upper West Region, Ghana. Majority of the respondents had some form of formal education. The age, marital status, education, occupation, gravity and family setup were found to be associated with knowledge of Hepatitis B infection and MTCT. Thus, there is urgent need to intensify efforts of health staff to educate expectant mothers. In addition, home education and outreach activities should be intensified on HBV infection as well as MTCT. Consequently, planning, implementation and execution of preventive activities, especially in the antenatal clinics should critically consider the social and demographic variations of mothers.

**Electronic supplementary material:**

The online version of this article (doi:10.1186/s12879-017-2490-x) contains supplementary material, which is available to authorized users.

## Background

Ghana is considered as endemic for hepatitis B infection with an estimated national prevalence of 10% [1–3]. Hepatitis refers to the inflammation of the liver. There are several types of hepatitis and its infectious causes include viral hepatitis A, B, C, D, E, G and other non-viral and non-infectious causes. The Hepatitis B virus (HBV) is most common in developing countries and causes infection which can lead to death resulting from liver cirrhosis, cancer and liver failure [4]. Universally, an average of 2 billion people are infected with the HBV and well over 240 million of this number have developed chronic liver infection, with an estimated annual mortality of 1 million [5]. Sub-Saharan Africa, comparatively, has the most predominant prevalence rate of chronic hepatitis B infection among its mature population, of about 5 to 10% [6]. A major route of HBV transmission, especially in endemic areas is Mother-To-Child Transmission (MTCT), also known as the perinatal transmission [7]. MTCT of HBV refers to a positivity of the hepatitis B surface antigen or the HBV-DNA at 6 to 12 months of a child’s life born to an infected mother [8]. Higher percentage of the global chronic carrier cases of HBV, especially in children, is due to MTCT. There is about 80–90% chance of developing chronicity and possible liver complications later in life [9]. There are few reported studies on the knowledge of HBV infection among expectant women [10], particularly in developing countries where illiteracy still remains a major challenge. In spite of the fact that, infection with the HBV remains a universal public health concern, not much is known about its epidemiology for the duration of pregnancy in sub-Saharan Africa [11]. Evidence from Kenya, Cameroon, and Nigeria, recorded limited knowledge of the occurrence of HBV infection among expectant mothers [11–14], though a simple clinical test for HBV infection is available in many countries.

In Ghana, there is a compulsory component of the array of medical tests required of every expectant woman who visits a health facility for antenatal services including clinical test for HBV infection. Thus, it is expected that all pregnant women will be screened for HBV surface antigen and also have some knowledge of HBV disease, especially the perinatal route of transmission [10]. With the existence of a potent vaccine against HBV infection, it is expected that good knowledge of the disease coupled with strict adherence to antenatal and postnatal policy measures would lead to the establishment of HBV-free generation. The Upper West Region is among the poorest regions with relatively high illiteracy rate in the country. The study therefore hypothesized low knowledge of HBV infection among expectant mothers, and relatively high carrier rate. Thus, the objective of this study was to assess the knowledge of expectant mothers’ on Mother-To-Child Transmission of viral Hepatitis B in Upper West Region, Ghana.

## Methods

### Study area

Two (2) of the eleven (11) districts in the Upper West Region of Ghana were selected for the study: namely; Wa Municipality and Lawra District. The Wa Municipality lies in the South Western part of the Upper West Region between Longitudes 9° 32″ W and 10° 20″W and Latitudes 1° 40″N and 2°45″N while the Lawra District is located between longitudes 2° 25″W and 2°45″W and Latitudes 10° 20″ N and 11° 00″N [15]. The Wa Municipality and Lawra District have an estimated total land area of 579.86 and 483.6 km^2^ respectively. Projecting from the 2010 Population and Housing Census, an estimated population of 107,214, comprising of 52,996 males and 54,218 females, and 50,703, comprising 23,628 males and 27,075 females have been recorded for Wa Municipality and Lawra District respectively. The Wa Municipality had an estimated population density of 185 persons/km^2^ and the population of Women In Fertility Age (WIFA) was 29,396 whiles the Lawra District had a population density of 105 persons/km^2^ and the population of WIFA was 13,185 as at December 2014 [16, 17]. The Wa Municipality has been sub-divided in six (6) Sub-Municipals with a total of 26 government health facilities including Community-based Health Planning and Services (CHPS) and 4 private facilities. However, there were 22 active government and 4 private health facilities that offered Antenatal Care (ANC) services as at December 2014 [16]. The Lawra District on the other hand has been sub-divided into five (5) Sub-Districts with a total of 16 government health facilities and 1 private health facility which provide ANC services [17]. The following is a summary of the 6 Sub-Municipals for the Wa Municipality and the 5 Sub-Districts from the Lawra District and their respective number of active ANC health facilities:

### Study design

This was a descriptive cross-sectional study conducted in the antenatal clinic service facilities in Wa the Municipality and Lawra District from April to May 2014. The study population included all expectant mothers in the study area who were registered as ANC clients during the period of the study. Information on socio-demographic and obstetric characteristics of the respondents were collected as well as information on their general knowledge of the HBV infection and disease before proceeding to assess their specific knowledge of MTCT. The input variables included the socio-demographic and obstetric characteristics of the expectant mothers: age, educational background, occupation, family setup, average monthly income, marital status, rural-urban residence, gravidity and parity. The output variable included the knowledge on MTCT of HBV infection. For instance, an expectant mother’s obstetric characteristic of gravidity can directly influence her knowledge of the HBV infection and disease through the ANC education and counseling services.

### Sample size determination

In accordance with [18], the following logical arithmetic procedure N = {Z^2^ *(PQ)/d^2^} was used to compute the desired sample size for the study. Where *N* is the desired sample size, *Z* is the confidence level of 95%, *P* is the prevalence rate (19.0% and 15.1%) of hepatitis B in the Wa Municipality and Lawra Districts respectively. *Q* is a constant computed as 1-P, and *d* is (5%) the set of margin of error. Therefore, for the Wa Municipality, giving hepatitis B prevalence of 19.0%, the sample size was calculated as:

$$ \frac{\mathrm{N}={\mathrm{Z}}^2\times \mathrm{PQ}}{{\mathrm{d}}^2}\frac{=\left\{{(1.96)}^2\ast \left(0.19\left(1-0.19\right)\right)\right\}}{0.05^2}\frac{=0.5912}{0.0025}=236 $$

Assuming a 5%, non-response rate, 12 respondents were added to arrive at a sample size (248) in the Wa Municipal.

Similarly for the Lawra District, assuming hepatitis B prevalence of 15.1%, the sample size was calculated as:

$$ \frac{\mathrm{N}={\mathrm{Z}}^2\times \mathrm{PQ}}{{\mathrm{d}}^2}\frac{=\left\{{(1.96)}^2\ast \left(0.15\left(1-0.15\right)\right)\right\}}{0.05^2}\frac{=0.489804}{0.0025}=199 $$

Assuming a 5%, non-response rate, 11 respondents were added to the sample size (210) in the Lawra district. These resulted in an overall sample size of 458.

### Sampling procedures

A three-stage random sampling procedure was used to select the sub-districts, health facilities (HFs) and individual respondents for the study. At the first stage of sampling, all sub-districts in Wa Municipality and Lawra District were included in the study. That is, six sub-districts in Wa Municipality and five sub-districts in Lawra District. At the second stage of sampling, the HFs were selected per sub-district respectively. Selection of the HFs was done at the sub-district levels. For each sub-district, list of all HFs where ANC services are available was compiled and through the lottery method, one HF was randomly selected from the lists respectively. However, due to the fact that majority of women visit the Wa Regional and Lawra District Hospitals respectively, the two were purposively included in the study. This is because the two hospitals are situated in the capital towns of areas that are of comparatively high population densities in their respective districts. The Municipal hospital also serves as the regional hospital where as the hospital in Lawra sub-district also doubles as the district hospital. As such, they are well resourced with staff and equipment such as HBV test equipment, ultrasound scan machines, etc. as compared to the other health facilities. In all, thirteen (13) HFs were selected for the study; seven (7) HFs in the Wa Municipality and six (6) HFs in the Lawra District (see Table 1).

The third stage involved the selection of respondents at the HF level using the quota sampling method. For each of the study areas, a calculated sample size was allocated among the selected HFs in proportion to their past ANC attendance. All selected HFs were visited by the field team during ANC days. As a routine at the facility level, pregnant women who attend ANC for services usually sit in queues on the basis of first-come-first-served. They then file in one after the other to consult the ANC nurse for assessment and counseling where necessary. Each time a facility was visited, respondents were recruited by consecutive sampling, as and when the pregnant women arrived for their scheduled ANC services. Pregnant women who arrived earlier than the research team were usually prompted to participate in the study after going through their ANC schedule if they had some time to spare. This was done to ensure that every pregnant woman who attended the facility for ANC services at the time of this study was given opportunity to participate in the study. This recruitment procedure was usually done until the quota for that facility was met, if not, subsequent visits were made until the quota for that facility was exhausted. At the ANC level in each health facility, the study protocol was clearly explained to the expectant mothers in the English language and/or in a local dialect for those who did not understand the English language. At the individual level, their voluntary participation in the study was sought and only those who agreed to participate were recruited. Post-natal mothers and other women who were at the facilities but were not confirmed ANC registrants were excluded from this study.

Research Assistants were recruited among university students with health background from the study areas. This was to ensure that the recruits had adequate understanding of the HBV disease concept as an infectious communicable disease. They were trained on the purpose of the study, the methodology, socio-cultural norms and questionnaire administering techniques. A pre-testing of the questionnaire was done before the main data collection started. The study used a semi-structured questionnaire to collect the information from the pregnant women at the ANC facilities. In addition, the antenatal cards of expectant women were also sought and assessed to confirm the information provided. To avoid misclassification bias in designing the questionnaire for the study, the questions on knowledge were purposefully extracted from peer reviewed journal articles on the general knowledge of HBV infection and also on specific knowledge on MTCT of HBV. Further review and classification assistance was sought from a clinical professor. Further information on data collection can be found in Additional file 1.

### Data analysis

The data was entered into Microsoft Office Excel for Windows version 2010 and checked for data inaccuracies and errors to ensure consistency. Though the study sampled 458 respondents, only 450 were included in the analyses following the data cleaning process; the 8 respondents were found to be incomplete and were excluded from the analysis. It was then exported to Statistical Package for Social Sciences (SPSS) software version 20.0 for Windows for analysis. The data was analyzed and presented in the form of frequencies, percentages, proportions and tabulations.

## Results

### Socio-demographic characteristics of respondents

Table 2 shows the socio-demographic characteristics of the respondents. A total of 450 respondents were included in the analysis and majority (54.0%) were within the ages of 25–35 years. The proportion of respondents who had some level of formal education was 62.4% with nearly 20.0% higher in Wa Municipality compared to Lawra District. Approximately, 76.2% of the respondents were multigravida with similar results in Wa and Lawra Districts. In terms of respondents’ occupational status, majority (56.0%) were unemployed. However, majority (56.2%) of the employed respondents were in the Wa Municipality as against 30.0% in the Lawra District. Polygamy was generally practiced by 33.6% of the respondents though monogamy was predominant (58.0%). In terms of income, nearly all (95.3%) expectant mothers earned an average monthly income of either GHC500 or less.

**Table 1:** ANC Health facilities in Wa Municipality and Lawra District in 2014

Municipal/District	Sub-Municipal/Sub-district	Number of ANC HFs	Number sampled
Wa Municipality	Wa Central Sub	5	2
	Kambali Sub	4	1
	Bamahu Sub	4	1
	Charia Sub	2	1
	Charingu Sub	5	1
	Busa Sub	3	1
Lawra District	Lawra Sub	7	2
	Babile Sub	3	1
	Eremon Sub	2	1
	Domwini Sub	3	1
	Zambo Sub	2	1
Total ANC HFs	40		13

Source: Extracts from [16, 17]

**Table 2:** Socio-demographic characteristics of respondents

Variable	Total		Wa Municipality		Lawra District	
	N	%	N	%	N	%
Age group (years)						
≤24	137	30.4	64	26.7	73	34.8
25–35	243	54.0	140	58.3	103	49.0
>35	70	15.6	36	15.0	34	16.2
Total	450	100.0	240	100.0	210	100.0
Marital Status						
Married	382	84.9	209	87.1	173	82.4
Not Married	68	15.1	31	12.9	37	17.6
Total	450	100.0	240	100.0	210	100.0
Occupation						
Unemployed	252	56.0	105	43.8	147	70.0
Employed	198	44.0	135	56.2	63	30.0
Total	450	100.0	240	100.0	210	100.0
Educational level						
Educated	281	62.4	171	71.2	110	52.4
Uneducated	169	37.6	69	28.8	100	47.6
Total	450	100.0	240	100.0	210	100.0
Gravidity						
Primidgravida	105	23.3	57	23.8	48	22.9
Multigravida	343	76.2	182	76.2	161	76.6
Total	448	99.5	240	100.0	209	99.5
Average monthly income						
≤ GHC500	429	95.3	227	94.5	202	96.2
GHC501–1000	17	3.8	9	3.8	8	3.8
> GHC1000	4	0.9	4	1.7	0	0.0
Total	450	100.0	240	100.0	210	100.0
Family Set-up						
Polygamous	151	33.6	89	37.1	62	29.5
Monogamous	261	58.0	132	55.0	129	61.4
Other	37	8.2	18	7.5	19	9.0
Total	449	99.8	239	99.6	210	100.0

Table 3 presents the number of respondents that were recruited from the 13 ANC health facilities in the respective sub-municipals and sub-districts. Two ANC facilities were sampled in the Wa Sub-Municipality: the Wa regional hospital in the Wa Municipality was a constant and the Wa Central HC which was randomly sampled. Similarly in the Lawra district, two ANC facilities were sampled under the Lawra Sub-district: the Lawra district hospital was a constant and the Lawra Sub HC which was randomly sampled.

**Table 3:** List of health facilities sampled and the quota of sample size

Sno	Sub-District	Health Facility	ANC Attendance	% of Total Attendance	Quota Sampled
Wa Municipality					
1	Kambale Sub	Kambale HC	697	5.64	14
2	Bamahu Sub	Bamahu HC	407	3.30	8
3	Charingu Sub	Charingu HC	282	2.28	6
4	Busa Sub	Busa HC	279	2.26	5
5	Wa Sub	Wa Reg. Hospital	8942	10.87	180
6	Wa sub	Wa Central HC	1341	72.50	27
7	Charia Sub	Dobile CHPS	392	3.18	8
Total			12,340	100.00	248
Lawra District					
8	Eremon Sub	Eremon HC	107	8.89	19
9	Lawra Sub	Lawra Dist. Hosp	715	59.43	125
10	Lawra Sub	Lawra Sub HC	28	2.33	5
11	Babile Sub	Babile Polyclinic	194	16.13	34
12	Domwini Sub	Domwini HC	42	3.49	7
13	Zambo Sub	Zambo HC	117	9.73	20
Total			1203	100.00	210

Quota sample is the product percentage of respective ANC attendance and the Sample Size

Table 4 shows the respondents’ knowledge about hepatitis B infection and disease. In total, less than half of the respondents, 46.2% (208) knew about hepatitis B infection and its disease. Knowledge of the infection and disease was lower in Lawra District (40.1%) compared to Wa Municipality (51.6%). Overall, respondents’ knowledge of liver damage/cirrhosis as a consequence of hepatitis B infection was 47.1%. The knowledge was higher in Lawra District (50.0%) compared to Wa Municipality (45.0%). About half of the respondents, 49.0% (219) of respondents knew that the HBV can be transmitted through blood and/or blood products. Respectively in the Lawra District and Wa Municipality, 35.2% (74) and 60.4% (145) of the respondents knew that HBV infection could occur through contact with blood or blood products. In total, 42.8% (193) of the respondents knew that unprotected sex could lead to HBV transmission. The awareness was higher in Wa Municipality compared to Lawra district (57.0% vs. 26.7%) (See Table 4).

**Table 4:** Knowledge of Hepatitis B infection and disease

General knowledge of HBV infection & disease	Overall	Wa Municipality	Lawra District
	*N* (%)	*N* (%)	*N* (%)
HBV is more Infectious than HIV/AIDS	132 (29.3)	62 (25.8)	70 (33.3)
HBV can lead to liver damage/cirrhosis	212 (47.1)	107 (44.6)	105 (50.0)
There’s blood test for HBV infection	341 (75.8)	199 (82.9)	142 (67.6)
HBV can be transmitted via blood/products	219 (48.7)	145 (60.4)	74 (35.2)
HBV infection is possible via sharing of food, drinks, witchcraft, etc.	40 (8.9)	21 (8.8)	19 (9.0)
The HBV can be transmitted through unprotected sex with an infected person	193(42.8)	137(57.0)	56(26.7)
An HBV infected infant may not show any sign and symptoms	349 (77.5)	189 (78.8)	160 (76.2)
Knowledge of your HBV status?	349 (77.5)	131 (54.6)	48 (22.9)
Percentage Knowledgeable	208 (46.2)	124 (51.6)	84 (40.1)
Total number of respondents	450^a^	240^a^	210^a^

^a^Percentages do not add up to 100% due to multiple responses

### Respondents’ knowledge of mother-to-child transmission of HBV

Table 5 shows the average knowledge level of Mother-To-Child Transmission of viral hepatitis B among respondents was 34.7%. This is comparatively higher in the Wa Municipality (43.3%) than Lawra District (24.8%). In some specific cases, only 1.8% of respondents knew that breast milk poses no verified risks of transmitting HBV, and 76.2% knew that an HBV carrier expectant mother can pass on the virus to her child during or after delivery. In total, 39.3% of the respondents also knew that a child who is not vaccinated against the HBV can still be infected after birth from an infected mother. Meanwhile, 56.7% of the respondents in Wa Municipality as compared to 19.5% in Lawra District knew that a child who is not vaccinated against HBV can still be infected after birth from an infected mother. However, 16.7% of the respondents agreed that HBV vaccine was safe, protects against HBV infection, and poses no harm to the unborn baby. This was similar among respondents in Wa Municipality and Lawra District.

**Table 5:** Expectant mothers’ knowledge of mother-to-child transmission of HBV

Knowledge about MTCT of HBV	Correct response only *N* (%)		
	Total N (%)	Wa Municipality	Lawra District
An HBV infected Mother can infect her baby in the uterus (intrauterine) before birth	343(76.2)	202 (84.2)	141(67.1)
Breast milk can transmit HBV	8(1.8)	3(1.2)	5(2.4)
Newborns are too young to contract HBV from infected mothers	177(39.3)	136 (56.7)	41(19.5)
The HBV vaccine is harmful to the unborn	75(16.7)	51 (21.1)	24 (11.4)
HBV infection of the child cannot occur after the child is born to an infected mother.	175(38.9)	128 (53.3)	47(22.4)
Average Knowledge about MTCT (sum of % N/5)	156(34.7)	104 (43.3)	52 (24.8)
Total number of respondents	450	240^a^	210^a^

^a^Percentages do not add up to 100 due to multiple responses

### Respondents’ socio-demographic characteristics and their knowledge about MTCT of HBV

Table 6 presents finding on how the socio-demographic characteristics of respondents were associated with their knowledge about MTCT of HBV infection in the study area. Each socio-demographic factor was analyzed using binary regression model to determine its relationship with their knowledge about MTCT of HBV infection. In the combination of the two districts (Wa Municipal and Lawra District), married (*P* = 0.007 and OR = 2.13), uneducated (*P* = 0.007 and OR = 1.83), unemployed (P = <0.001 and OR = 2.46) and primigravida (*P* = 0.008 and OR = 1.92) had statistically significant association with their general knowledge of MTCT of HBV compared to unmarried, educated, employed and multigravida respondents.

**Table 6:** Socio-demographic determinants of knowledge about MTCT of HBV using Binary logistics regression analysis

	Overall		WMHD		LDHD	
	*P*-value	OR(95%CI)	*P*-value	OR(95%CI)	*P*-value	OR(95%CI)
Age						
≥ 30 years		1.0		1.0		1.0
≤ 30 years	0·472	1·24(0·69, 2·20)	0·404	1·60(0·53, 4·82)	0·13	0·56(0·27, 1·19)
Marital status						
Not Married		1.0		1.0		1.0
Married	0·007	2·13(1·23, 3·69)	0·002	0·27(0·12, 0·62)	0·48	0·77(0·37, 1·60)
Education						
Educated		1.0		1.0		1.0
Not Educated	0·007	1·83(1·18, 2·84)	0·977	0·99(0·46, 2·12)	0·017	0·49(0·27, 0·88)
Occupation						
Employed		1.0		1.0		1.0
Not Employed	<0·001	2·46(1·54, 3·95)	0·122	0·58(0·29, 1·16)	0·015	0·42(0·21, 0·85)
Family Setup						
Monogamous		1.0		1.0		1.0
Polygamous	0·965	1·01(0·65, 1·57)	0·015	2·44(1·19, 4·99)	0·092	0·59(0·32, 1·09)
Gravidity						
Multigravida		1.0		1.0		1.0
Primigravida	0·008	1·92(1·82, 3·10)	0·003	2.99(1·43, 6·24)	0·272	1·45(0·75, 2·84)
Income						
≥ GHS500		1.0		1.0		1.0
≤ GHS500	0·998	5·37(0·00, 0·00)	0·999	3·24(0·00, 0)	0·999	8·38(0·00, 0)

When it was analyzed by separate districts, the Wa Municipality found married (*P* = 0.02 and OR = 0.27), polygamous family setup (*P* = 0.015 and OR = 2.44) and primigravida (*P* = 0.003 and OR = 2.99) were statistically significant associated with their knowledge of MTCT of HBV compared to unmarried, monogamous family setups, and multigravida. While in the Lawra District, only uneducated (*P* = 0.017 and OR = 0.49) and unemployed (*P* = 0.015 and OR = 0.42) had statistical significant evidence of association with their knowledge of MTCH of HBV compared to educated and employed (see Table 6).

## Discussion

Expectant mothers’ knowledge of HBV infection is very critical in eliminating the transmission cycle of the virus in newborns. In contrast, majority of the respondents in this study had some formal education above primary level and yet their knowledge was generally low. Generally, the study revealed a relatively low general knowledge of the HBV infection and disease (46.2%) and MTCT of HBV (34.7%) among the expectant mothers in the study area. Specifically, less than half (39.3%) of the overall respondents knew that newborns to HBV infected mothers are at risk of contracting the HBV. Few (1.8%) of the mothers correctly said that the transmission could possibly occur via breast milk, especially if the mother is also tested positive of the Hepatitis B envelop antigen (HBeAg). Majority of the respondents did not know their HBV status or their spouse’s. This finding should be a major concern for the health authority in the two districts. Expectant mothers with high HBeAg values could be transmitting the infection to their babies in ignorance. The observation of relatively low knowledge made in this study supports that of [19] who found that overall knowledge among their respondents regarding hepatitis B was poor and inaccurate. It however differed from a study conducted by Adeyemi and colleagues who found that majority of their study respondents didn’t have formal education with responding low level of knowledge about HBV transmission [14].

Majority of the respondents were within the ages of 20 to 35 years, which is similar to findings reported in other studies [14, 20–22]. They found that majority of their respondents were within a youthful age of 25 to 35 years. Many studies assessing the knowledge and prevalence of HBV infection, especially among pregnant women affirm that, women <30 years of age are at a higher risks of being infected with the HBV [10–14, 23, 24]. This has been attributed to unsafe sexual practices among such people which predisposes them to sexually transmitted infections [25].

Literacy rate among respondents was generally high as majority had some form of formal education above primary school level. This result could be due to the fact that Ghana has an overall literacy rate of 56.3% and female literacy rate of about 47% [26] while the Lawra District has a female literacy rate of 59.7% [27]. The Wa Municipality is a relatively urban setting with a comparatively educated population in that, approximately 70% of persons who are older than 14 years and living in urban areas of Ghana are literate [26]. In addition, there is about 44% Secondary School enrolment participation ratio among females in Ghana [28], with relatively higher proportions in urban areas. The interplay of these factors possibly account for the relatively high literacy rate among the respondents since majority of the sample were resident in the central sub-districts of the two areas.

Majority of the respondents earned monthly income of 500 Ghana cedis (GHC 500) or less on an average - approximately 125 United States Dollars (USD 125.00) and above the national poverty line of GHC 106 per month per individual [29]. This might imply that on an average, majority of the expectant mothers in the Wa Municipality may be unable to afford basic health care services such as blood screening for HBV, and the cost of the HBV vaccination for themselves as individuals. The average household density as revealed by the study was five persons in the Wa Municipality and seven in Lawra District. This does not include indirect dependents as it is usually the case in most developing countries. The financial demands on these mothers are relatively high, thereby affecting their health seeking behaviors [30]. Maternal access to comprehensive blood screening test and vaccination cover for the whole family against HBV infection could be challenging, especially in the comparatively larger homes.

The association between maternal age distribution and their knowledge of MTCT of HBV particularly in the Wa Municipality, is similar to the findings that maternal age below 35 years is a strong predictor of knowledge of MTCT of HBV [11, 13, 14, 31]. Though majority of the respondents were educated, no significant association was found between educational status and knowledge level of MTCT of HBV in the Wa Municipality, contrary to reports by [10, 24]. However in the Lawra District, there was an association between respondents’ educational background and their knowledge of MTCT of HBV. This indicates that more expectant mothers in the Lawra District who were educated had correct knowledge about MTCT of HBV unlike their uneducated colleagues. This is also similar to the finding of many other studies around the globe [10, 11, 14, 20, 32].

The finding with regards to the association between respondents’ marital status and their knowledge of MTCT of HBV in the Wa Municipality in particular, contradicts the finding by [19] who reported no association between marital status and knowledge about HBV infection. The current findings predict that married respondents were knowledgeable than their unmarried colleagues could be a quantitative difference since there were many married respondents who were also younger in age than the unmarried. In the case of the Lawra District however, there was no association between respondents’ marital status and their knowledge about MTCT of HBV which rather agrees with the report of [19].

The study also found an association between respondents’ family setup and their knowledge of MTCT of HBV infection among the respondents in the Wa Municipality unlike in the Lawra District. This is an important indicator for the current study because many studies that have explored knowledge about HBV infection among expectant mothers did not discuss this relationship. In 2014, a knowledge assessment study among Cameroonian pregnant women captured the indicator of family setup but did not find any significant association between that and knowledge of HBV among the respondents [11].

The occupational status of our respondents in the Lawra District was also associated with their knowledge of MTCT of HBV unlike in the Wa Municipality. The probable reason for this relationship could be linked to respondents’ educational background. The educational background of respondents in the Lawra District was also found to be significantly associated with their knowledge of MTCT of HBV. This contradicts the findings of [13, 20, 29], and yet agrees with other findings in similar studies around the globe [30, 31].

The obstetric gravidity of the respondents in the Wa Municipality was also significantly associated with their knowledge of MTCT of HBV infection. This could result from the assumption that the expectant mothers in the Wa Municipality were regular to their ANC schedules as expected and benefited from the regular education provided at the ANC clinics. This finding conforms to the finding of [19] who reported significant relationship between the knowledge of their respondents and their obstetric gravidity. The absence of association between respondents’ gravidity and their knowledge as in the Lawra District agrees with reports by [31] which did not also find any significant linkage between gravidity and knowledge among Nigerian expectant mothers. Unlike the finding of [20], the finding of no significant relationship between expectant mothers’ educational background and their knowledge about MTCT of HBV infection agrees with [11]. Generally, there was no significant association between the respondents’ monthly income and their knowledge of MTCT of HBV infection as also reported by [32].

## Conclusion

The knowledge of MTCT of HBV among expectant mothers in the Upper West Region was low in general. Majority of the respondents had some form of formal education. The age, marital status, education, occupation, gravity and family setup were found to be significantly associated with knowledge of Hepatitis B infection and MTCT in the Upper West Region. Thus, there is urgent need to intensify the efforts of health staff to educate more expectant mothers. In addition, home education and outreach activities should be intensified on HBV infection as well as MTCT. Consequently, planning, implementation and execution of preventive activities, especially in the antenatal clinics should critically consider the social and demographic variations of mothers.

## Additional file

Additional file 1: HBV Paper for BMC Inf.D - QUESTIONNAIRE 22.05.2017. (DOCX 21 kb)

## Abbreviations

ANC: Antenatal Care
CHPS: Community-based Health Planning and Services
GHC: Ghana Cedis
GHS: Ghana Health Service
HBeAg: Hepatitis B ‘envelop’ Antigen
HBV: Hepatitis B Virus
HBV-DNA: Hepatitis B Virus Deoxyribonucleic Acid
HC: Health Center
HF: Health Facility
HFs: Health Facilities
HIV/AIDS: Human Immunodeficiency Virus/Acquired Immune Deficiency Syndrome
LDHD: Lawra District Health Directorate
MTCT: Mother-to-Child Transmission
OR: Odds Ratio
SPSS: Statistical Package for Social Sciences
UDS: University for Development Studies
USD: Unite States Dollars
WIFA: Women in Fertility Age
WMHD: Wa Municipal Health Directorate
