# Supplementary material for: Assessing the knowledge of expectant mothers on mother–to-child transmission of viral hepatitis B in Upper West region of Ghana
Source: BMC Infect Dis. 2017 Jun 12;17:416. doi: 10.1186/s12879-017-2490-x (PMC5469103; doi:10.1186/s12879-017-2490-x)
Supplement: Additional file 1: — HBV Paper for BMC Inf.D - QUESTIONNAIRE 22.05.2017. (DOCX 21 kb) [file 12879_2017_2490_MOESM1_ESM.docx]

## THESIS QUESTIONNAIRE

UNVERSITY FOR DEVELOPMENT STUDIES

SCHOOL OF ALLIED HEALTH SCIENCES

(DEPARTMENT OF COMMUNITY HEALTH)

**MPHIL. COMMUNITY HEALTH AND DEVELOPMENT**

**Topic:** Assessing the Knowledge of Expectant Mothers on Mother-To-Child Transmission of Viral Hepatitis B in Upper West Region of Ghana.

**Interviewer’s Code:…………. Respondent’s Number……….**

**District:……… Cluster/Sub-Dist.:………………… HF/ANC Name:……………**

PREAMBLE

This comparative quantitative study seeks to assess the knowledge level of Expectant mothers about mother-to-child transmission of Hepatitis B Virus (*wurduru* or *popaal/Saoriibaalong*) in the Upper West Region. The information you would provide shall solely be used for an academic purpose and your absolute confidentiality shall be maintained. However, your agreement or disagreement to partake in this study is purely voluntary and at your discretion; in either way you would not be penalized in any way.

**NOTE**: *Please carefully choose your response to each question (that would be posed to you) as you deem right.*

1. **SOCIODEMOGRAPHY**
2. Age (complete years)….
3. Marital status? A. Married [ ] B. Widowed [ ] C. Co-habiting [ ] D. Separated [ ]
4. Highest Educational level completed? A. None [ ] B. Primary [ ] C. JHS [ ] D. SHS [ ] E. Tertiary [ ]
5. Main Occupation? A. Farming [ ] B. Civil/Public Service [ ] C. Private Business [ ] D. Student [ ] E. Unemployed [ ]
6. Religious Affiliation? A. Islam [ ] B. Christianity [ ] C. ATR [ ] D. Other (State)………..
7. Residential status? A. Resident [ ] B. New Immigrant [ ] C. None [ ]
8. Family set up? A. Polygamous [ ] B. Monogamous [ ] C. Other (State…………)
9. Household Size (number of persons feeding from same pot)? State number………………
10. Average Monthly Income ………..
11. **Obstetric Characteristics:**
12. Gravidity status: A. Primigravida [ ] B. Gravida 2 [ ] C. Gravida 3 [ ] D. Multigravida [ ]
13. Parity status: A. Nullipara [ ] B. Para 1 [ ] C. Para 2 [ ] D. Para 3 [ ] E. Multipara [ ]
14. **General Knowledge about HBV Infection and Disease**
15. Hepatitis B Virus is more infectious than the HIV. A. Yes [ ] B. No [ ] C. Don’t Know [ ]
16. HBV Infection can lead to liver damage (cirrhosis) or cancer. A. Yes [ ] B. No [ ] C. Don’t Know [ ]
17. There is a blood screening test for hepatitis B infection. A. Yes [ ] B. No [ ] C. Don’t Know [ ]
18. Hepatitis B Virus can also be transmitted through blood or blood products. A. Yes [ ] B. No [ ] C. Don’t Know [ ]
19. One can get infected with Hepatitis B disease through sharing of drinks, food with infected person or through witchcraft. A. True [ ] B. False [ ] C. Don’t know [ ]
20. Hepatitis B Virus can be transmitted through unprotected sex with an infected person. A. Yes [ ] B. No [ ] C. Don’t Know [ ]
21. An infected child may not show any signs and symptoms until late in life A. True [ ] B. No [ ] C. Don’t Know [ ]
22. Do you know your HBV status? A. Yes [ ] B. No [ ]
23. **Knowledge about mother-to-child Transmission of HBV**
24. An HBV infected pregnant woman can infect her unborn child too. A. Yes [ ] B. No [ ] C. Don’t Know [ ]
25. A child born to an infected mother can still be infected through breast feeding A. Yes [ ] B. No [ ] C. Don’t know [ ]
26. New born babies are too young to get hepatitis B infection from positive mothers A. True [ ] B. False [ ] C. Don’t know [ ]
27. The Mother-to-Child transmission of HBV can only occur before birth. A. Yes [ ] B. No [ ] C. No [ ]
28. The HBV vaccine during pregnancy is harmful to the unborn baby A. True [ ] B. False [ ] C. Don’t know [ ]
29. Hepatitis B Infection cannot occur once the child is born (to an infected mother) A. True [ ] B. False [ ] C. Don’t Know [ ]
30. **Knowledge about Preventing MTCT of HBV**
31. The following are all appropriate ways to help prevent the infection/spread of the Hepatitis B Virus.

**a.** Screening and vaccination A. True [ ] B. False [ ] C. Don’t know [ ]

**b.** Childhood HBV vaccine A. True [ ] B. False [ ] C. Don’t know [ ]

**c.** There is a hepatitis B vaccine available for uninfected but non-immunized adults. A. Yes [ ].

**e.** The unborn child can be protected from hepatitis B infection by drinking some herbs A. True [ ] B. False [ ] C. Don’t know [ ]

**f.** If an HBV infected mother is on treatment there is no need vaccinating the child at birth against the disease A. True [ ] False [ ] C. Don’t know [ ]

**g.** Post-Exposure prophylaxis (Mother)/Treatment A. True [ ] B. False [ ] C. Don’t know [ ]

B. No [ ] C. Don’t Know [ ]

**h.** Avoid sharing of and pricks from hypodermic objects A. True [ ] B. False [ ] C. Don’t know [

**F. HBV Status/Medical History**

1. Premarital/Pre-pregnancy HBV check-up? A. Yes [ ] B. No [ ]
2. Family history of HBV infection? A. Yes [ ] B. No [ ] C. Don’t know [ ]
3. Do you know your husband’s HBV status? A. Yes [ ] B. No [ ]
4. HBsAg Status (*Observation from ANC Booklet*). A. Reactive [ ] B. Non-Reactive [ ] C. Untested/Undeclared [ ]

*Your honest participation and time spent are greatly appreciated.*

**Interviewer’s Attention!!!**

Please read over all the questions to ensure that all are fully answered. Repeat and/or rephrase any questions you might have skipped for clarity of understanding and ensure they are answered fully before allowing the respondent to leave.

**Interviewer’s Signature and (Initials)………………………………**

Thank you
